# Serpentinization-Associated Mineral Catalysis of the Protometabolic Formose System

**DOI:** 10.3390/life13061297

**Published:** 2023-05-31

**Authors:** Arthur Omran, Asbell Gonzalez, Cesar Menor-Salvan, Michael Gaylor, Jing Wang, Jerzy Leszczynski, Tian Feng

**Affiliations:** 1 Department of Chemistry, University of North Florida, Jacksonville, FL 32224, USA; 2 Department of Geosciences, University of South Florida, Tampa, FL 33620, USA; 3 Departmento de Biologia de Sistemas, Universidad de Alcala, 28805 Alcala de Henares, Spain; 4 Analytical Sciences, Small Molecules Technologies, Bayer U.S., Saint Louis, MO 63167, USA; 5 Department of Chemistry, Physics and Atmospheric Sciences, Jackson State University, Jackson, MS 39217, USA

**Keywords:** serpentinization, chemical garden, hydrothermal environments, formose reaction, prebiotic chemistry, metabolism, chemical evolution, chemical complexity, systems chemistry

## Abstract

The formose reaction is a plausible prebiotic chemistry, famed for its production of sugars. In this work, we demonstrate that the Cannizzaro process is the dominant process in the formose reaction under many different conditions, thus necessitating a catalyst for the formose reaction under various environmental circumstances. The investigated formose reactions produce primarily organic acids associated with metabolism, a protometabolic system, and yield very little sugar left over. This is due to many of the acids forming from the degradation and Cannizaro reactions of many of the sugars produced during the formose reaction. We also show the heterogeneous Lewis-acid-based catalysis of the formose reaction by mineral systems associated with serpentinization. The minerals that showed catalytic activity include olivine, serpentinite, and calcium, and magnesium minerals including dolomite, calcite, and our Ca/Mg-chemical gardens. In addition, computational studies were performed for the first step of the formose reaction to investigate the reaction of formaldehyde, to either form methanol and formic acid under a Cannizzaro reaction or to react to form glycolaldehyde. Here, we postulate that serpentinization is therefore the startup process necessary to kick off a simple proto metabolic system—the formose protometabolic system.

## 1. Introduction

### 1.1. Formose Reaction or Formose System?

Living systems require complex chemical reactions that are interwoven together [1,2,3]. In order to understand this simply we look to simple experiments in prebiotic chemistry. The formose reaction is a classic experiment from the 1800s predating the field of prebiotic chemistry. Later on, it was interpreted as prebiotic chemistry, analogous to experiments like the Miller-Urey experiment. The formose reaction consists of one reactant—formaldehyde—reacting under basic conditions to form a complex mixture of sugars [4,5,6,7,8,9,10]. These sugars range from two and three carbon sugars to pentoses, hexoses and larger sugars, branched chain sugars, ketoses, and aldoses. This reaction is prebiotically plausible, being that formaldehyde forms under UV light in planetary atmospheres and could be delivered by meteors [10,11,12,13,14]. Classically, the reaction is catalyzed by calcium ions [4,5,6,7,8,9,10]. The formose reaction is autocatalytic, meaning that the reaction can catalyze itself. This means that its products, such as glycolaldehyde, help to drive the reaction on, further lowering the activation energy cost to produce products. To date, the discussion of the formose reaction has almost exclusively been about sugar production. Ribose is also a product of the formose reaction. Hence, prebiotic chemists have focused on the formose reaction in the hopes of finding a system that could facilitate the evolution of genetic materials. Our interest in the formose reaction is not predicated on the production of sugars.

Under alkaline hydrothermal conditions, most of the sugar produced by the formose reaction forms breakdown products such as lactic acid [9]. These simple organic acids are associated with metabolism [9]. Our focus on the formose reaction is the production of metabolically relevant simple organic acids. Throughout this work, we shall pose the idea that this is a distinct system—the protometabolic formose system—that incorporates the classical formose reaction and yields simple acids based on its environmental conditions.

### 1.2. Serpentinization Environments: Land and Sea

This system requires energy and a formaldehyde source. Furthermore, it needs alkaline conditions. Which geological settings and geochemical processes could provide these necessities? One excellent example of geochemistry that could support burgeoning prebiotic chemistry would be the serpentinization system [15,16,17,18]. This system occurs when olivine or pyroxene containing rocks are hydrolyzed by water, releasing heat and hydrogen gas; they then form serpentinite rocks/minerals [15,16,17,18]. Any carbon dioxide trapped within the serpentinization environment undergoes reductions by hydrogen gas, ultimately forming methane, with many intermediates such as formic acid, methanol, and formaldehyde [18]. Therefore, serpentinizing environments that contain carbon dioxide could plausibly yield formaldehyde [18]. Carbon monoxide trapped within the system would form formaldehyde, methanol, and ultimately methane as well [18].

The serpentinization process itself thus provides a gradient of different redox states of carbon, including formaldehyde. Sources for formaldehyde include the photochemical production of formaldehyde in the atmosphere raining down, meteoric delivery from space, and electrochemical production [10]. These sources provide both formaldehyde and energy from impacts, lightning, radiation, or serpentinization. The process of serpentinization makes the surrounding aqueous environment alkaline [15,16,17,18].

We note this in environments such as the Lost City Hydrothermal Field. In these environments, great chimney structures form out of calcium and magnesium carbonates and hydroxides [19,20,21]. These precipitation mounds/chimneys are naturally occurring examples of chemical gardens. A chemical garden is a tubular precipitation structure made of ionic salts formed under alkaline conditions in silicate- or hydroxide-based solutions [4,22,23,24]. So, we note environments fostered by serpentinization that are alkaline and thus sequester calcium and magnesium together as mineral carbonates and hydroxides [21]. The hydroxide ions are easy to justify as they are in the basic solution. Carbonates form due to interactions with water and carbon dioxide and are more prevalent under basic pH environments (hydroxide ions consume protons in solution, favoring more bicarbonate and carbonate ions at equilibrium). 

This phenomenon is not suited only to deep-sea environments. Serpentinization occurs on land as well [16]. While we do not see precipitation chimneys on dry land, the ground water and any pooled water that forms when it rains become alkaline and sequester carbon dioxide. The carbon dioxide forms carbonates and precipitates with calcium and magnesium [25,26,27]. Incidentally, the rain would then provide the water for serpentinization in the first place, on the land. Additionally, as a pond dries down, it could become more alkaline, as it would decrease in volume. As the pond hydrates, salts could hydrolyze and resolubilize, adding cations back into the solution. There are many benefits afforded to prebiotic chemistry in a dry land system by wet–dry cycling.

### 1.3. Lewis-Acid-Based Catalysis and Heterogeneous Catalysis

Protometabolic systems might be so simple that they predate proteins/enzymes. For these systems, mineral catalysts would serve as the primordial enzymes. Minerals can be classified as heterogeneous catalysts—solid-state catalysts present in a solution but not dissolved into it [28]. Lewis-acid catalysis occurs when a metal-based Lewis acid acts as an electron pair acceptor to increase the reactivity of the reactants [28,29,30,31,32]. Therefore, when metal ions in solution or in a heterogeneous catalyst coordinate reactants and lower the activation energy for them to form products with, we can refer to them as Lewis-acid heterogenous catalysts [28,32]. In this work, we use minerals associated with serpentinization as Lewis-acid heterogenous catalysts. 

### 1.4. Proposal

Thinking backwards from our understanding of serpentinization systems, one may envision a system where the planet through geochemistry provides the energy, reducing the power, reactants, and conditions necessary to foster a simple organic system of proto metabolites. If the protometabolic system persists and repeatedly produces products, changes in the environment would serve as selective pressures on the system. Hence, chemical evolution would occur with the system. We see a necessity for the formose protometabolic system in nature, fostered by serpentinization, in land and sea environments. The system should be catalyzed by minerals in the environment. We address this through DFT calculations and by looking at a plausible mechanism for the initial step of the formose reaction. We then look for catalysis by various minerals associated with serpentinization systems and for the products formed. 

## 2. Materials and Methods

### 2.1. DFT Calculations

The Minnesota density functional M06-2X [33,34,35] was applied for the present investigation. The basis set used was the standard polarization functions and the diffuse functions augmented valence triple-zeta basis set 6-311+G(*d*,*p*) [36]. The Barone–Tomasi polarizable continuum model (PCM) [37] with the standard dielectric constant of water (*ε* = 78.39) was applied to simulate the solvated environment of an aqueous solution. The force constants were determined analytically in the analysis of harmonic vibrational frequencies for all of the complexes. The DFT method included in the GAUSSIAN 09 program [38] was used for all computations. 

All the calculations were performed at the M06-2X/6-311+G(*d*,*p*) level. We looked at the gas phase and liquid phase thermodynamic parameters (Gibb’s free energy) for the reaction of formaldehyde to either form methanol and formic acid under a Cannizzaro reaction, or to react to form glycolaldehyde in the first step of the formose reaction. Additionally, we computed changes in activation energy barriers in a second set of calculations. These calculations were carried out at the M06-2X/6-311+G(*d*,*p*) level with a PCM model (in a water solvent).

### 2.2. Mineral/Chemical Gardens Synthesis and Analysis

Salt pellets were made by compressing CaCl_2_ and MgCl_2_ (both by Fischer) in a 1:1 ratio by hand. Pellets were placed into a vial containing 1 M sodium meta-silicate (Fischer) solution and allowed to sink to the bottom of the vial. The 1 M sodium meta-silicate solution contained 100 mM Na_2_CO_3_ (Fisher). Chemical gardens took about 10 min to grow into the solution at room temperature (25 °C). Chemical gardens were removed from solution, washed with DI water three times to remove external silicates, and allowed to dry overnight. The chemical gardens and minerals, including olivine, serpentinite rocks, and magnetite, were inspected via Raman Spectroscopy and powder X-ray diffractometry (XRD) on a BTX-402 Benchtop XRD and at 785 nm on an Enwave EZI-785-A2 Raman spectroscope. Data were analyzed using Crystal Sleuth Software (RRUFF project 2008 edition).

### 2.3. Formose Reactions Synthesis and Analysis 

Formaldehyde solutions were prepared by diluting concentrated formaldehyde with methanol solution, to a final concentration of 1 M formaldehyde containing 0.5 M methanol (Fischer). The methanol was an added stabilizer to the formaldehyde solution, to prevent the formation of paraformaldehyde. The solution was titrated with 1 M NaOH to a final pH of about 12.5. The solution was poured into borosilicate vials. Some solutions had 0.5 g of powdered mineral or chemical gardens added to them; others had calcium or magnesium chlorides or carbonates added to them as controls, and some vials had no salts or minerals added to them and served as negative controls. Each combination of reagents was tested in triplicate, with all solutions being heated in a hot water bath at 90 °C on a hotplate. Vials were heated for one hour and inspected optically and via time-point sampling with NMR.

After completion, mineral samples and chemical garden samples were filtered out of the solution and inspected via XRD to note any possible changes in structure or ratio. 

Solutions were filtered on 0.2 µm pore nylon syringe filters (Thermo Scientific) using 10 mL Hamilton syringes. That is, 0.5 mL of each sample was aliquoted into NMR tubes along with 100 mM 4,4-dimethyl-4-silapentane-1-sulfonic acid (DSS, Sigma-Aldrich) and 10% by volume D_2_O (Sigma-Aldrich). Solutions were characterized using ^13^C NMR and 1D proton magnetic resonance measurements with water suppression pulses on a Bruker Neo Advance 600 MHz NMR. Some solutions were spiked with 10 mM of organic acids, including glycolic acid, acetic acid, lactic acid, and formic acid, as described in previous work [9]. Data analyses were run on SpinWorks 4.0 or MNOVA software and SigmaPlot 10.0 software. 

## 3. Results

### 3.1. Cannizzaro Reaction DFT

**Scheme 1:**
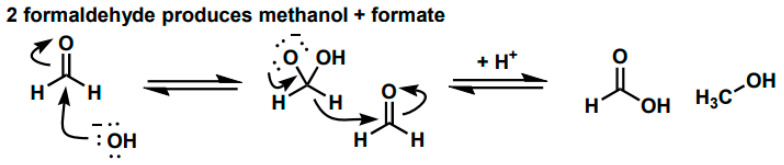
The Mechanism of the Cannizzaro Reaction of Formaldehyde.

Our calculations comparing the formose reaction to the Cannizzaro reaction (Scheme 1) show the predominance of the Cannizaro reaction under many circumstances. Here, we compare the Gibb’s free energy values (∆G) of the first step of the formose reaction (the reaction of formaldehyde to glycolaldehyde) to the Cannizaro reaction of formaldehyde (the formation of methanol and formic acid from formaldehyde) (Table 1).

Reaction 1: The Cannizzaro Reaction

CH_2_O + CH_2_O + OH^−^ → CH_3_OH + CHOO^−^

∆G1 = G(CHOO^−^) + G(CH_3_OH) − G(OH^−^) − 2G(CH_2_O)

Reaction 2: The first step of the formose reaction

2 CH_2_O → C_2_H_4_O_2_

∆G2 = G(C_2_H4O_2_) − 2G(CH_2_O)

The above results suggest that products of the Cannizzaro reaction would be more favored than those of the formose reaction. Both reactions prefer the gas phase to the water phase. A lower temperature would benefit both reactions in either the gas or water phase. The Cannizzaro reaction occurs in base conditions. Acidic conditions might benefit the first part of the formose reaction.

### 3.2. Materials Identification 

**Figure 1:**
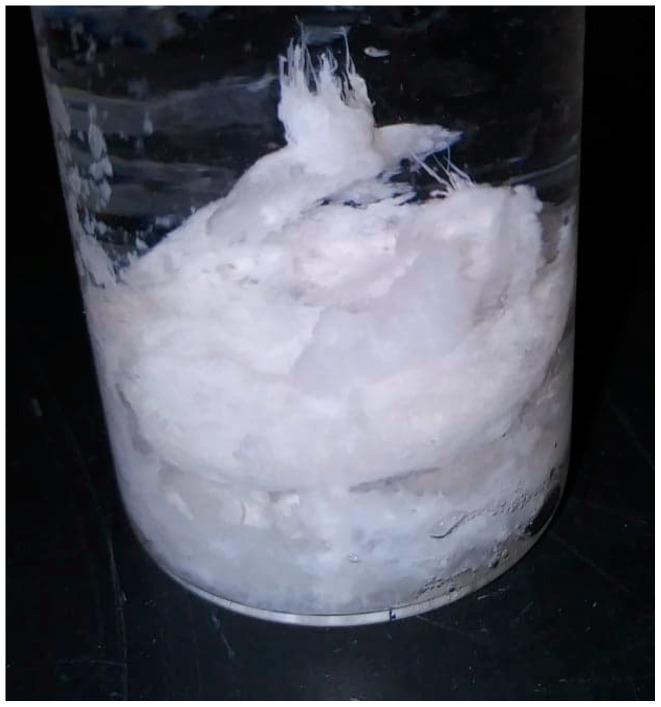
Ca/Mg-based classical chemical garden. The garden formed from a 0.25 g pellet of 1:1 CaCl_2_/MgCl_2_ submerged into a 1 M Na_2_SiO_3_ solutions that contained 100 mM Na_2_CO_3_.

Pellet chemical gardens formed over a period of 10 min, forming from 1:1 CaCl_2_/MgCl_2_ pellets in silicate solution (Figure 1). The pH of the solution was about 12 and it contained sodium carbonate. 

**Figure 2:**
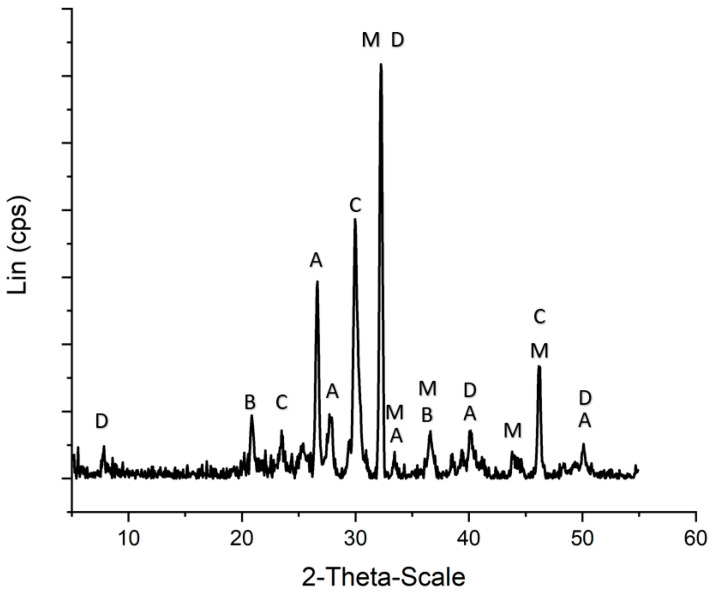
Powder X-ray diffraction pattern for a Ca/Mg-Based chemical garden. The garden is comprised of microcrystalline B = brucite R040077, C = calcite R040170, A = aragonite R040078, M = magnesite R040114, and D = dolomite R040030. The garden is also comprised of amorphous calcium and magnesium, carbonates, hydroxides, and silica. Base line calibrated with Crystal Sleuth software.

The chemical gardens were inspected using powder XRD and Raman Spectroscopy to investigate which minerals the crystalline regions were comprised of (Figure 2 and Figure S1). These minerals included dolomite, calcite, aragonite, magnesite, and brucite. The purity of the other minerals was verified spectroscopically as well (Figures S2–S5). All data were compared to standards on the RRUFF database [39]. Magnetite was not verified this way; it was, however, purchased at 99% purity from Sigma Aldrich.

### 3.3. Formose Products 

Products of the formose reaction were identified using NMR, including ^13^C NMR, spiked-in experiments, and ^13^C/^1^H HSQC (Figures S7–S12). The formaldehyde solutions we worked with were pure and newly bought stock solutions from Fischer; we also checked the concentrations for accuracy with previously developed methods [40]. These results verify the results of our previous work [9]. Additional controls were run, showing that methanol and paraformaldehyde did not yield similar products when investigated in the same fashion that our formose reactions were (data not shown). Additional formose products were identified using glycosyl composition analysis (Table S1, Figures S13–S17). NMR and GCMS analysis indicated several small organic acids associated with metabolism. These included glycolic acid, lactic acid, formic acid, acetic acid, and oxalic acid (Table 2, Figures S8–S12). This was the first time oxalic acid has been detected in this system; it was not detected in our previous work [9] due to being proton-silent.

Glycosyl composition analysis indicated trace amounts of sugars, including glucose, mannose, xylose, and arabinose (Table S1, Figures S13–S17). There were only trace amounts of sugars detected compared to the other products, and sugar yields were extremely low due to sugars degrading in hot alkaline conditions. We verified lactic acid production from sugar breakdown by running a control of an alkaline 0.167 M glucose solution, and we observed similar products in that and other controls (Figures S19 and S20, Table S2).

In order to observe the expanded view of the alkaline formose reaction and its fuller link to metabolism, we note that most products in this system do not end in sugars, and based on the experimental conditions such as basicity and heat Cannizzaro products and tars, they seem inevitable.

### 3.4. Formose System Catalysis with DFT

Our system uses minerals and chemical gardens as heterogenous catalysts. The use of these catalysts and ions in solution as catalysts is an example of Lewis-acid-based catalysis. The reaction of formaldehyde to sugar is followed by a yellowing step; for this yellowing step to occur, sugars must be in solution and then degrade. We conducted multiple time-point experiments to demonstrate the catalytic properties of our minerals and chemical gardens.

In one experiment, we optically observed yellowing of solutions containing added minerals or no added minerals, and we compared average yellowing times (Figure S18). Solutions containing our chemical garden samples, calcium or magnesium salts, or serpentinite or olivine all reacted much faster than our control group (no minerals/salts added). Additionally, magnetite showed no catalysis (Figure S18). Interestingly, our chemical garden sample reaction time fell between calcium and magnesium chloride and calcium and magnesium carbonates; it is likely that the garden was leaching ions into the solution. 

**Figure 3:**
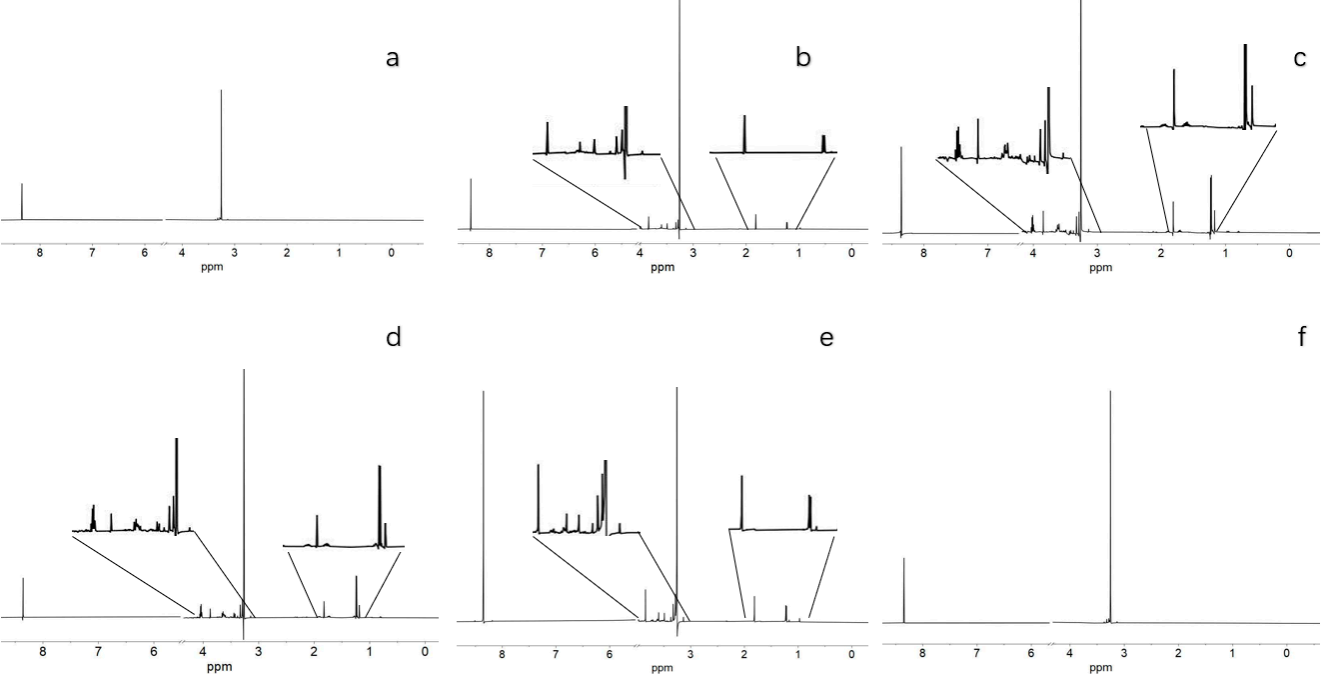
Time-Point Experiment. Samples extracted from ongoing reactions at 25 min, then placed on ice. (**a**) Reaction with no minerals or chemical gardens, only Cannizzaro products found. (**b**) Formose reaction with Ca and Mg CO_3_ powder. (**c**) Formose reaction with Ca/Mg-Silica Garden. (**d**) Formose reaction with olivine. (**e**) Formose reaction with serpentinite. (**f**) Formose reaction with magnetite, only Cannizaro products found. These samples were analyzed using NMR with water suppression, peaks at 1.3 and 4.1 ppm represent lactic acid, 1.9 ppm indicates acetic acid, 8.4 ppm formic acid, 3.9 ppm represents glycolic acid, and 3.2 ppm represents methanol.

In addition to faster reaction times in the presence of some of our mineral samples, we looked at product distribution at the 25 min time point to note which solutions had reacted and formed formose products. Here, we observe that the control group (no minerals/salts added) has not formed any formose reaction products, and neither did the magnetite added group (Table S2). At the 25 min time point, we did identify formose products for our chemical garden, olivine, serpentinite, calcium, and magnesium salt groups (Table 3, Figure 3). The control and the magnetite groups showed only Cannizaro products in solution. This was another indicator that some reactions were catalyzed by the added minerals.

Lactic acid, being a sugar breakdown product, is an indicator of the formose reaction and was used to compare formose output between experimental groups. In terms of comparing experimental groups, the highest production of lactic acid was observed in our Ca/Mg mineral-containing samples (around 0.5 int. %), whereas serpentinite and olivine showed the second-highest amount lactic acid (0.36 and 0.31 int. %, respectively). Magnetite and the no mineral group showed no lactic acid (Table 3).

**Scheme 2:**
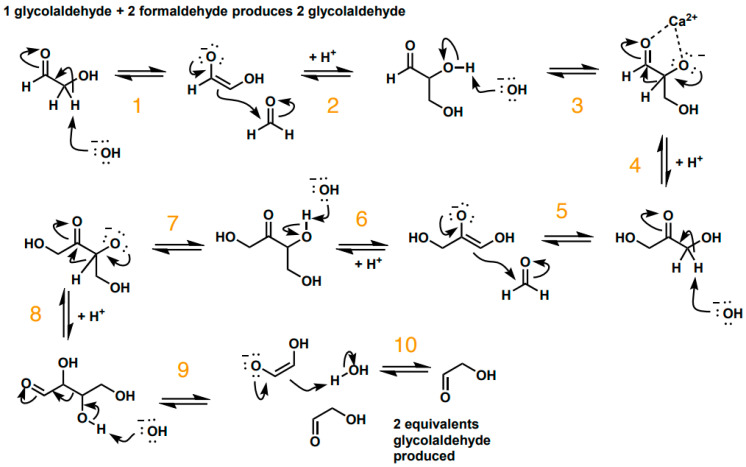
A Mechanism for the First step of the Formose Reaction with a Catalyst Present.

In order to further explain the observed catalytic process, we propose a mechanism for the formose reaction and use our DFT calculations to model what happens at each step of this mechanism (Scheme 2 and Table 4). Our mechanism addresses the slow part of the formose process—the formation of glycolaldehyde from formaldehyde.

Our DFT calculations show that the Ca^2+^ and Mg^2+^ ions help to lower the energy barriers for formaldehyde to form glycolaldehyde. Reaction steps 1, 2, and 3 all exhibit a decrease in the activation energy barrier from 53 kcal/mol to about 45 kcal/mol; this indicates a mechanism for the catalysis of the formose process by calcium and magnesium ions/minerals.

All the calculations were carried out at the M06-2X/6-311+G(*d*,*p*) level with a PCM model (solvent = water). From step 4 onwards (Scheme 2), Ca^2+^ and Mg^2+^ have little effects on the activation energies. For the entire reactions (from formaldehyde to glycolaldehyde), the results suggest that the rate-determining step should be Part 1 (steps 1–3). The steps in Part 2 (steps 4–10) have energy barriers that reveal that those reactions can be observed at room temperature. Glycolaldehyde might be helpful to condense formaldehyde into more glycolaldehyde. However, calcium and magnesium do not show many effects on accelerating the related reactions (as in steps 4–10). We did not show the null results for steps 4–10; we only showed the steps that had a change in activation energy, i.e., steps 1–3.

### 3.5. Mineral System Evolution 

**Figure 4:**
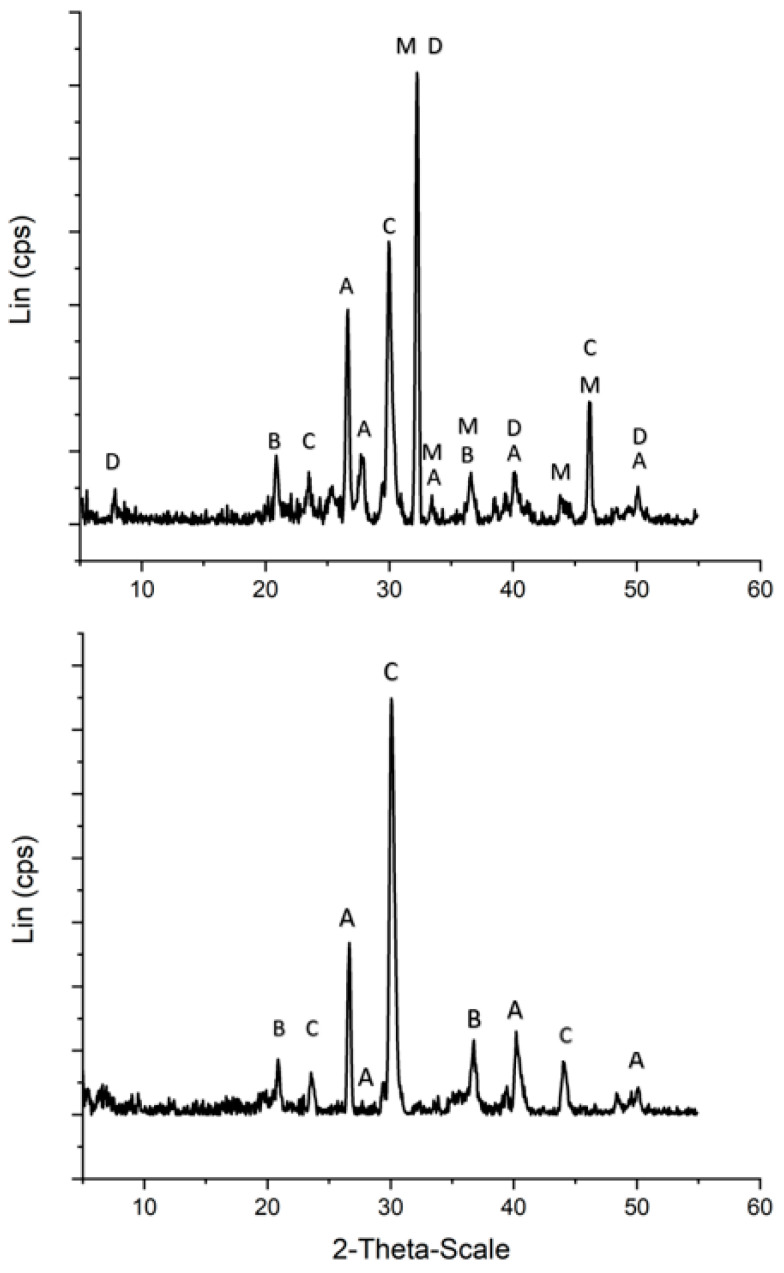
Powder X-ray diffraction pattern for a Ca/Mg-Based chemical garden (**top**) before being exposed to the formose reaction. Powder X-ray diffraction pattern for a Ca/Mg-Based chemical garden (**bottom**) after being exposed to the formose reaction. The garden is comprised of microcrystalline B = brucite, C = calcite, A = aragonite, M = magnesite, and D = dolomite. The garden is also comprised of amorphous calcium and magnesium, carbonates, hydroxides, and silica. Base line calibrated with Crystal Sleuth software.

In terms of understanding how our mineral systems changed during the reactions, we analyzed our minerals with powder XRD after our hydrothermal reactions were carried out. Our magnetite, olivine and serpentinite samples were not changed during our hydrothermal reactions (data not shown). The composition of the chemical garden sample did change, however (Figure 4). The garden composition changed due to hydrothermal alteration, from a composition rich in magnesium and calcium carbonates and hydroxides to a composition of primarily calcium carbonates such as calcite and aragonite. We replicated this change using hot water (instead of formaldehyde solution) (Figure S6), showing that the garden composition change was due to hydrothermal alteration and not the formose reaction.

## 4. Conclusions

### 4.1. Main Points Summarized 

In this work, we demonstrate that the Cannizaro process is the dominant process under many different conditions, thus necessitating a catalyst for the formose reaction under many different environmental circumstances. Our formose reactions produce primarily organic acids associated with metabolism and a protometabolic system, and have very little sugar left over. This is due to many of our acids forming from the degradation and Cannizaro reactions of many of the sugars produced during the formose reaction. Hence, our study mainly focuses on organic acids. We also show heterogeneous Lewis-acid-based catalysis of the formose reaction by mineral systems associated with serpentinization. The minerals that showed catalytic activity include olivine, serpentinite, and calcium, and magnesium minerals including dolomite, calcite, and our Ca/Mg-chemical gardens. This catalysis is due to the first three steps of the reaction, where formaldehyde reacts to form glycolaldehyde in the presence of calcium or magnesium. 

### 4.2. On the Potential Ubiquity of the Formose Proto-Metabolic Systems Due to Serpentinization

The origins and emergence of life need not be constrained to one environment or one system; any mineral-rich system could do, especially a hydrothermal system [41,42,43,44]. Another environment to consider would include potentially space-based environments that have a similar mechanisms of catalysis based on magnesium minerals [45]. The products in these systems may not necessarily be analogous to classical Butlerov formose-yielding sugars [9,44,45], but they are significant, nonetheless. 

The serpentinization of dry land systems can lead to pools or ponds of water in those systems becoming more alkaline, not only facilitating the pH needed for the formose reaction, but also the formation of Ca/Mg carbonate minerals needed for catalysis, as alkaline conditions absorb more CO_2_ (Scheme S1). Obviously, deep-sea systems could also exhibit this phenomenon, being that they can form atop serpentinizing crust and form mineral chimneys analogous to our chemical gardens (Scheme S2). These systems are rich with energy and produce catalysts for the formose reaction, and while conditions are not favorable for sugar stability, they do yield a protometabolic system of very simple organic acids, including lactic acid, formic acid, acetic acid, glycolic acid, and oxalic acid. It could be that serpentinization is how terrestrial planets and ice moons “breathe” life into protometabolic systems, the simplest example being the formose protometabolic system.

## Figures and Tables

**Table 1:** Free energy of Reactions (1) and (2) at different temperatures. ∆G(gas) are results in gas phase and ∆G(water) are results through PCM model (solvent = water). ∆G unit in kcal/mol, temperature in K. All the calculations were performed at M06-2X/6-311+G(*d*,*p*) level.

	250K	300K	350K	400K
∆G1(gas)	−73.3	−71.7	−70.0	−68.3
∆G2(gas)	−13.3	−11.4	−9.6	−7.8
∆G1(water)	−52.5	−50.9	−49.2	−47.5
∆G2(water)	−12.3	−10.5	−8.7	−6.9

**Table 2:** Products table of the formose and Cannizzaro products detected in our solutions.

Product.	Molecular Formula	Molecular Weight (amu)
Formic Acid	HCOOH	46.02
Acetic Acid	CH_3_CO_2_H	60.05
Glycolic Acid	C_2_H_4_O_3_	76.05
Lactic Acid	C_3_H_6_O_3_	90.07
Oxalic Acid	C_2_H_2_O_4_	90.03
Methanol	CH_3_OH	32.04
Arabinose	C_5_H_10_O_5_	150.13
Xylose	C_5_H_10_O_5_	150.13
Glucose	C_6_H_12_O_6_	180.15
Mannose	C_6_H_12_O_6_	180.15

**Table 3:** Product distributions of formose reactions after 25 min. Data are integral percentages of a set of data. * = no absorption due to their being no mineral to absorb to.

	Formic Acid	Glycolic Acid	Lactic Acid	Acetic Acid	Methanol	Adsorbed to Mineral
Dolomite Ca/Mg Chemical Garden	0.12	0.19	0.54	0.15	0.12	0.02
CaCl_2_ MgCl_2_	0.15	0.06	0.52	0.09	0.15	*
CaCO_3_ MgCO_3_	0.2	0.08	0.22	0.09	0.2	0.41
Serpentinite	0.24	0.07	0.36	0.08	0.24	0.01
Olivine	0.2	0.08	0.31	0.07	0.2	0.14
Magnetite	0.22	0	0	0	0.22	0.56
No Mineral	0.26	0	0	0	0.26	*

**Table 4:** Activation energy barriers associated with the formation of glycolaldehyde.

Formaldehyde Condensation to Glycolaldehyde					
Step		Reactants	Transition State	Products	Energy Barriers/kcal
1	2HCHO + H_2_O > CHOCH_2_OH	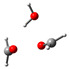	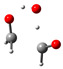	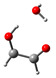	53
		1WTS2A	1WTS2	1WTS2B	
2	+Ca	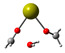	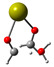	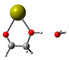	46
		1WCaTS2A	1WCaTS2	1WCaTS2B	
3	+Mg	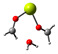	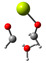	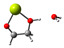	45
		1WMgTS2A	1WMgTS2	1WMgTS2B	

## Supplementary Materials

The following supporting information can be downloaded at: https://www.mdpi.com/article/10.3390/life13061297/s1, Figure S1: Raman Spectrum of Ca/Mg chemical garden. Spectrum taken with a 780 nm laser. Verified against RRUFF mineral database; Figure S2: Raman Spectrum of olivine sample. Spectrum taken with a 780 nm laser. Verified against RRUFF mineral database Figure S3: Raman spectrum of serpentinite. Spectrum taken with a 780 nm laser. Verified against RRUFF mineral database; Figure S4: Powder XRD diffraction pattern for olivine. 5–55-degree angle used. Verified against RRUFF mineral database; Figure S5: Powder XRD diffraction pattern for serpentine. 5–55-degree angle used. Verified against RRUFF mineral database; Figure S6: Hydrothermal alteration of the chemical garden. Hydrothermal alteration of the chemical garden occurs during the heating of the water for the formose process. During this process, the chemical garden transitions from a composition of calcite, magnesite, aragonite, brucite, and dolomite to a composition of mostly calcite, with some aragonite and Mg left; Figure S7: Concentrated Formose reaction. Chemical Garden facilitated reaction, dried overnight in the fume hood. Proton NMR with customized water suppression (noesygppr1d) technique on the Brucker Neo 600 with D2O Salt Locking. No peak at 3.2 ppm for methanol due to evaporative process; Figure S8: 13C NMR of a Ca/Mg-Based chemical garden facilitated formose reaction. Spectrum shows detection of glycolic acid, G, acetic acid, A, and lactic acid, L. Spectrum also indicates the presence of oxalic acid, Ox, which has yet to be detected in such studies due to it being proton silent; Figure S9: Formose solution with no spiked in products; Figure S10: Spiked in-experiment. Formose solution was spiked with glycolic acid, G, acetic acid, A, and lactic acid, L; Figure S11: Spiked in-experiment. Formose solution was spiked with glycolic acid, G, acetic acid, A, and lactic acid, L (the blue spectrum). This is overlayed onto a spectrum with no spiked in products (the black baseline spectrum). Spectrum shows clear detection of glycolic acid, G, acetic acid, A, and lactic acid, L. Spectral range from 0 to 4.3 ppm; Figure S12: HSQC of C13 (y axis) with H1 NMR (x axis) of a formose sample. Spectrum shows clear detection of glycolic acid, G, acetic acid, A, and lactic acid, L; Figure S13: Gas Chromatogram for the control formose reaction which contained no minerals or chemical gardens; Figure S14: Gas Chromatogram for the formose reaction which contained fragments of Ca/Mg-based chemical gardens. * Represent sugar like signatures that may be branched chain sugar species; Figure S15: Gas Chromatogram for the formose reaction which contained serpentinite minerals; Figure S16: Gas Chromatogram for the formose reaction which contained olivine minerals; Figure S17: Gas Chromatogram for the formose reaction which contained magnetite minerals; Figure S18: Observed reaction times for formose reactions in the presence of different minerals. The time it took each reaction to yellow, n = 3. The chemical garden group was statistically insignificant from the soluble Ca/MgCl_2_ group; Figure S19: CaCl_2_ MgCl_2_ (all aqueous) formose catalyzed solution proton NMR; Figure S20: Sugar break down control. 0.167 Glucose solution heated at 90 C for 30 min at pH. 12.5 proton NMR. We see the same peaks for lactic acid as we do in our formose reactions; Table S1: Detected sugar values. Mass and Mol % of the glycosyl residues identified for the formose reaction dried samples; Table S2: Product distributions in formose reactions after 25 min. Data are from integral percentages; Scheme S1: A Model for a Proto Metabolic Formose System on dry land; Scheme S2: A Model for a Proto Metabolic Formose System at a hydrothermal vent.
