# Supplementary material for: Serpentinization-Associated Mineral Catalysis of the Protometabolic Formose System
Source: Life (Basel). 2023 May 31;13(6):1297. doi: 10.3390/life13061297 (PMC10303151; doi:10.3390/life13061297)
Supplement: Supplementary file 1 [file life-13-01297-s001.zip › life-2400688-supplementary.pdf]

Supplemental Materials

Arthur Omran[1,2,*], Asbell Gonzalez [1], Cesar Menor-Salvan [3], Michael Gaylor [4], Jing Wang [5], Jerzy Leszczynski [5] and Tian Feng [2]

1 Department of Chemistry, University of North Florida, Jacksonville, FL 32224, USA

2 Department of Geosciences, University of South Florida, Tampa, FL 33620, USA

3 Departmento de Biologia de Sistemas, Universidad de Alcala, 28805 Alcala de Henares, Spain

4 Analytical Sciences, Small Molecules Technologies, Bayer U.S., Saint Louis, MO 63167, USA

5 Department of Chemistry, Physics and Atmospheric Sciences, Jackson State University, Jackson, MS 39217, USA

* Correspondence: arthur.omran@unf.edu

Table of Contents Page Number

1 Additional Methods 3

2 Additional Results 4

Figure S1 Raman Spectrum of Ca/Mg chemical garden 4

Figure S2 Raman Spectrum of olivine sample 4

Figure S3 Raman Spectrum of serpentinite 5

Figure S4 Powder XRD diffraction pattern for olivine 5

Figure S5 Powder XRD diffraction pattern for serpentine 6

Figure S6 Hydrothermal alteration of the chemical garden 7

Figure S7 Concentrated formose reaction proton NMR 8

Figure S8 C13 NMR of a Ca/Mg-Based chemical garden facilitated formose reaction 9

Figure S9 Formose solution with no spiked in products. 10

Figure S10 Spiked in-experiment. 10

Figure S11 Spiked in-experiment. Overlay 11

Figure S12 HSQC of C^{13} (y axis) with H^1 NMR (x axis) of a formose sample 11

Table S1 Detected sugar values. Mass and Mol % of the glycosyl residues 12

Figure S13 Gas Chromatogram for the control formose reaction 12

Figure S14 Gas Chromatogram for the formose reaction with Ca/Mg chemical gardens 13

Figure S15 Gas Chromatogram for the formose reaction which contained olivine minerals 13

Figure S16 Gas Chromatogram for the formose reaction which contained serpentinite minerals. 14

Figure S17 Gas Chromatogram for the formose reaction which contained magnetite minerals. 14

Figure S18 Observed reaction times for formose reactions in the presence of different minerals 15

Table S2 Product distributions in formose reactions after 25 minutes. 15

Figure S19 $CaCl_2$ $MgCl_2$ (all aqueous) formose catalyzed solution proton NMR. 16

Reaction Scheme S1: A model for a protometabolic system on dry land 16

Reaction Scheme S2: A model for a protometabolic system at a hydrothermal vent 17

Figure S20 Sugar break down control. 0.167 Glucose solution heated at 90 C for 30 minutes at pH. 12.5 proton NMR. We see the same peaks for lactic acid as we do in our formose reactions 17

1 Additional Methods

GCMS of Formose Samples to Detect Trace Sugar Species

Glycosyl composition analysis was performed by combined gas chromatography/mass spectrometry (GC/MS).Per-O-trimethylsilyl (TMS) derivatives of monosaccharide methyl glycosides were produced from the sample by acidic methanolysis. The dried formose sample (1.8 mg) with inositol (internal standard, 20 µg) was heated with methanolic HCl for 18 h at 80 °C. After cooling to room temperature and removal of the solvent under a stream of nitrogen, the samples were treated with a mixture of methanol, pyridine, and acetic anhydride for 30 minutes. The solvents were evaporated in a fume hood overnight. The samples were derivatized with Tri-Sil (Pierce) at 80 °C for 30 min. GC/MS analysis of the TMS methyl glycosides is performed on an Agilent 7890A GC, using a Supelco Equity-1 fused silica capillary column (30 m × 0.25 mm ID). The mixture of derivatized sample was analyzed by GC-MS and per-O-trimethylsilyl derivatives of monosaccharide residues run alongside the sample and used for quantification of each monosaccharide residue. The peak integration was used for quantification.

2 Additional Results

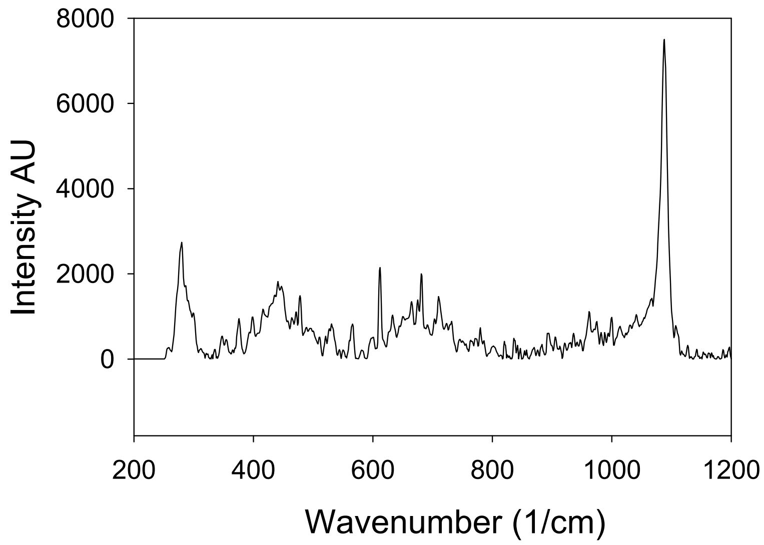

Figure S1. Raman Spectrum of Ca/Mg chemical garden. Spectrum taken with a 780 nm laser. Verified against RRUFF mineral database.

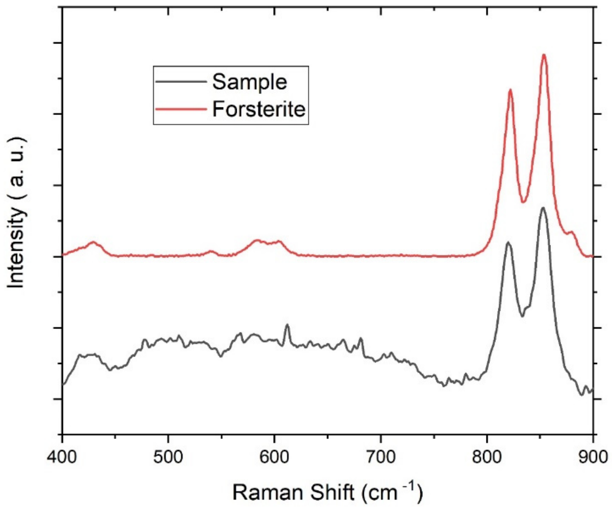

Figure S2. Raman Spectrum of olivine sample. Spectrum taken with a 780 nm laser. Verified against RRUFF mineral database.

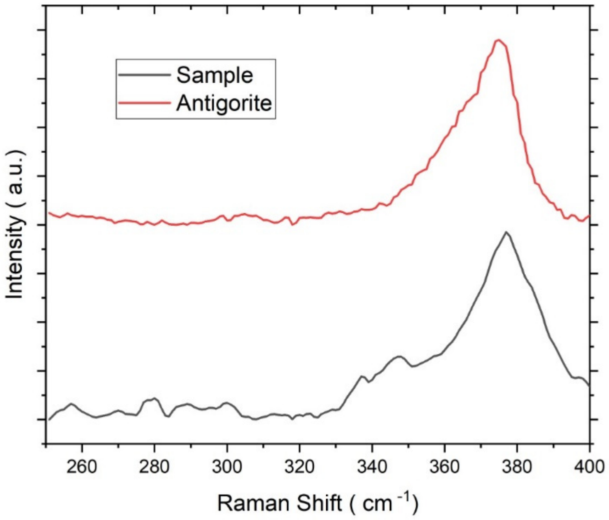

Figure S3. Raman spectrum of serpentinite. Spectrum taken with a 780 nm laser. Verified against RRUFF mineral database.

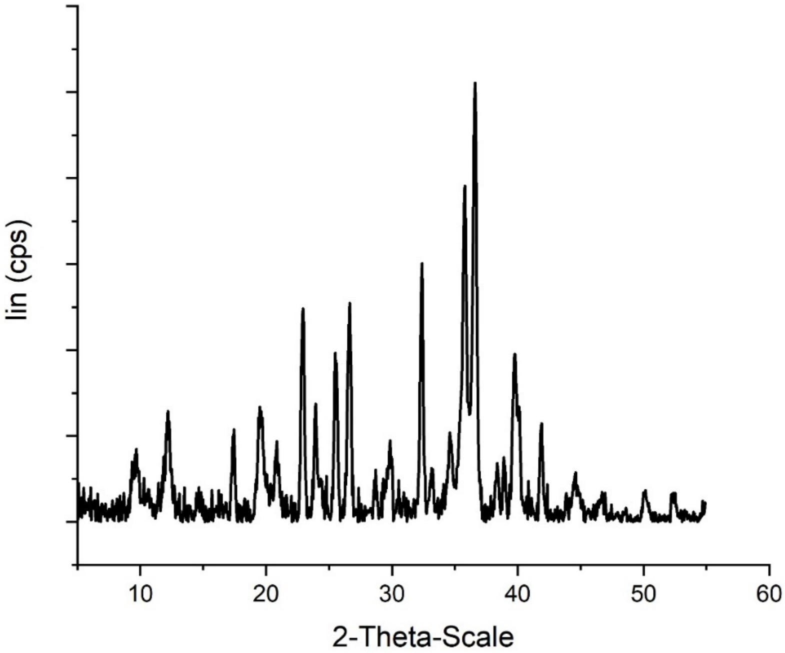

Figure S4. Powder XRD diffraction pattern for olivine. 5 – 55-degree angle used. Verified against RRUFF mineral database.

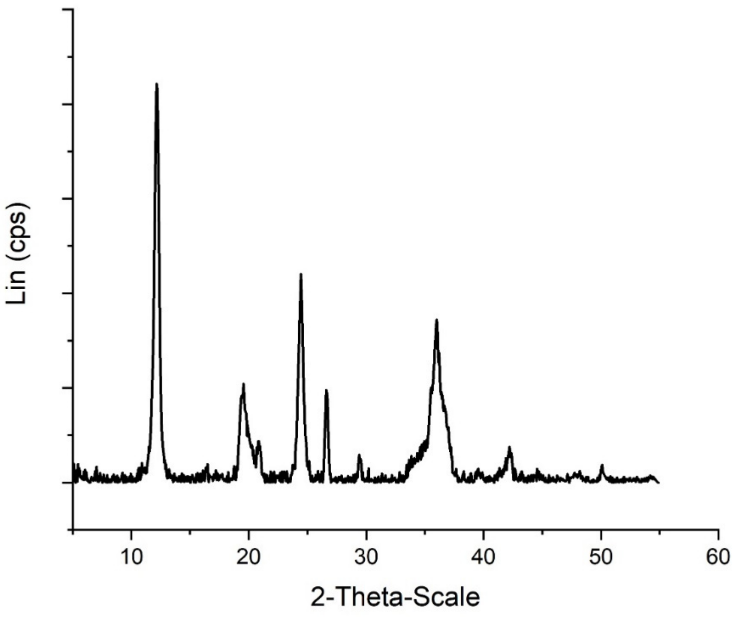

Figure S5. Powder XRD diffraction pattern for serpentine. 5 – 55-degree angle used. Verified against RRUFF mineral database.

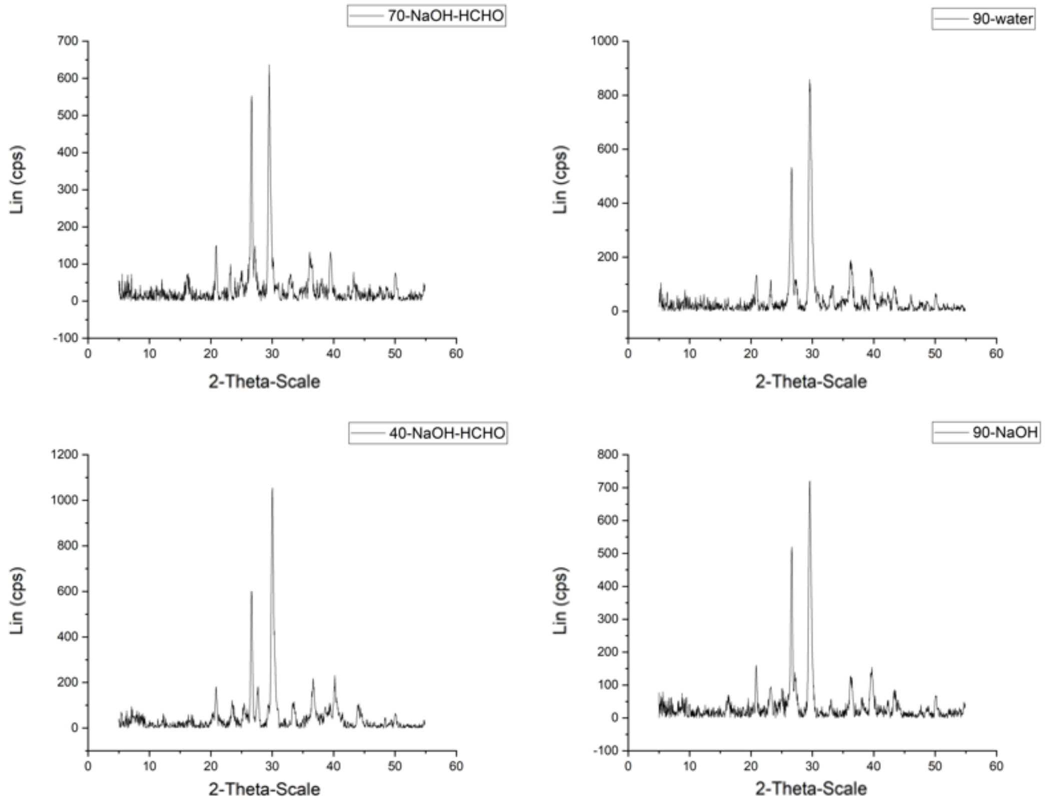

Figure S6. Hydrothermal alteration of the chemical garden. Hydrothermal alteration of the chemical garden occurs during the heating of the water for the formose process. During this process, the chemical garden transitions from a composition of calcite, magnesite, aragonite, brucite, and dolomite to a composition of mostly calcite, with some aragonite and Mg left.

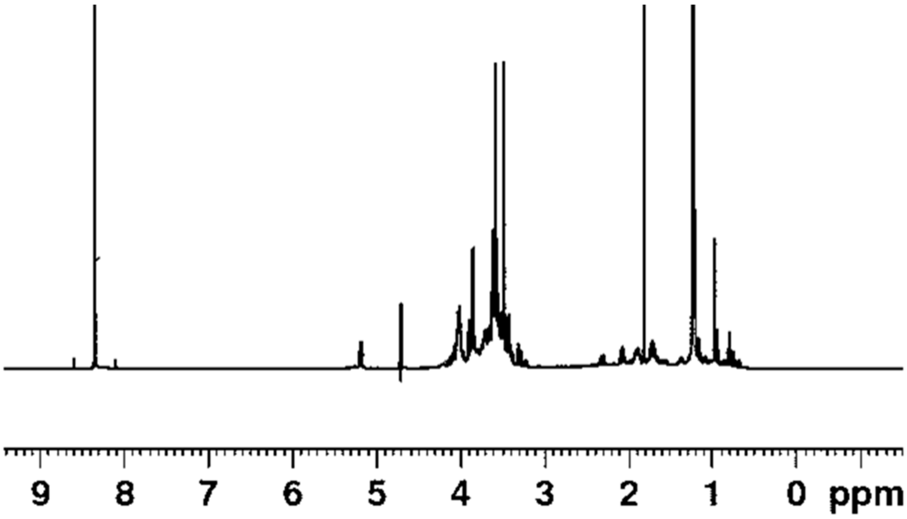

Figure S7. Concentrated Formose reaction. Chemical Garden facilitated reaction, dried overnight in the fume hood. Proton NMR with customized water suppression (noesygppr1d) technique on the Brucker Neo 600 with D_2O Salt Locking. No peak at 3.2 ppm for methanol due to evaporative process.

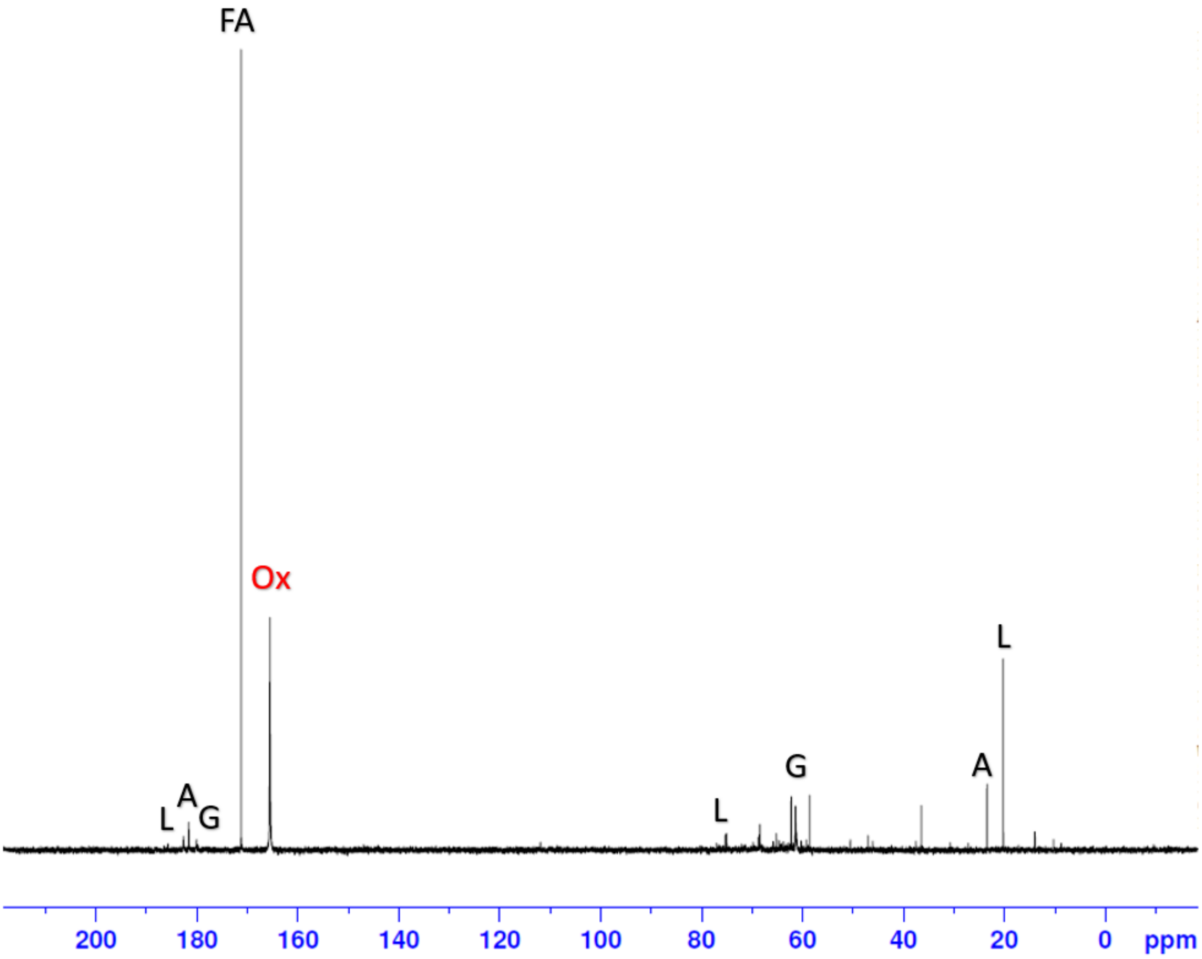

Figure S8. ^{13}C NMR of a Ca/Mg-Based chemical garden facilitated formose reaction. Spectrum shows detection of glycolic acid, G, acetic acid, A, and lactic acid, L. Spectrum also indicates the presence of oxalic acid, Ox, which has yet to be detected in such studies due to it being proton silent.

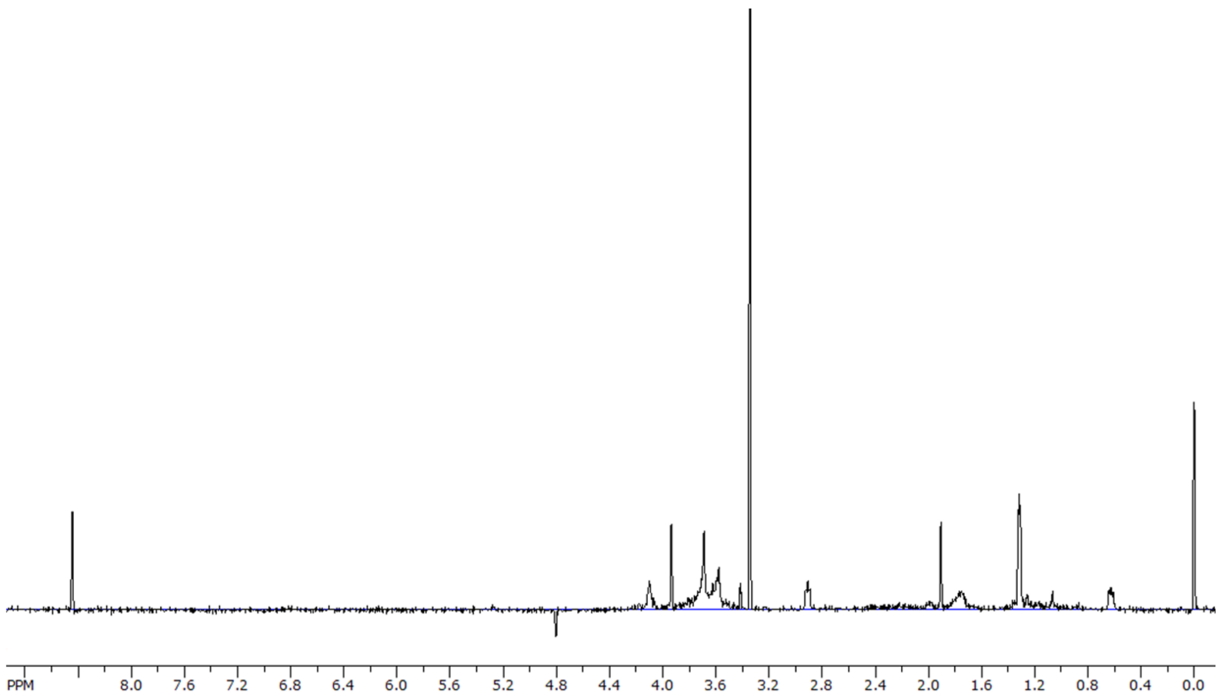

Figure S9. Formose solution with no spiked in products.

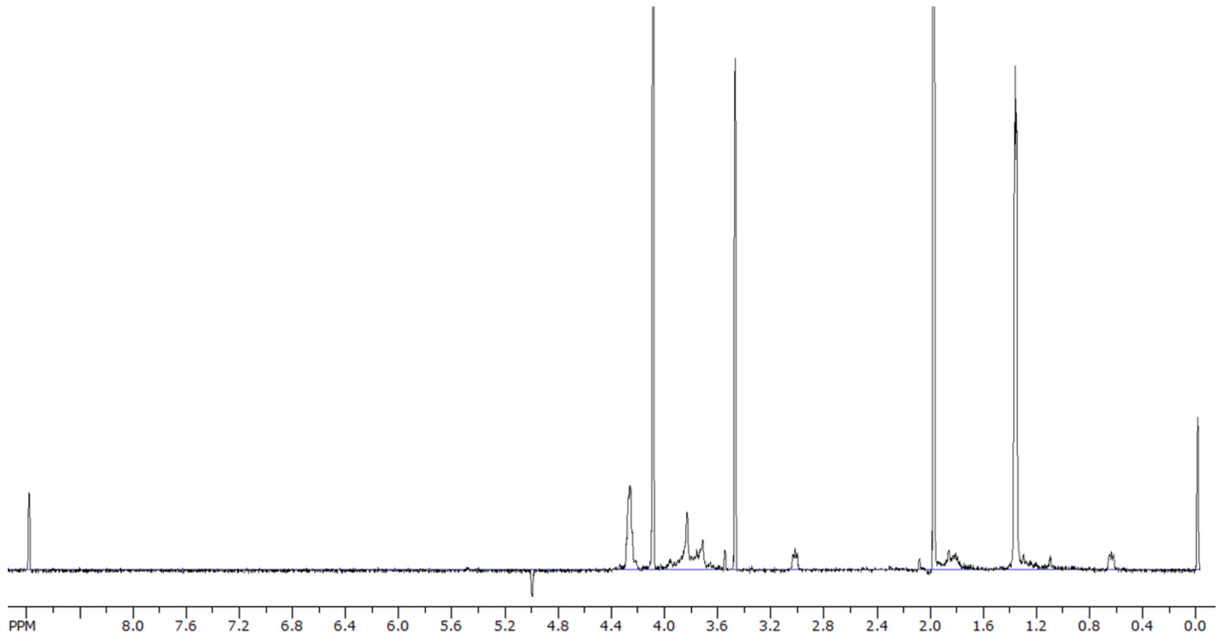

Figure S10. Spiked in-experiment. Formose solution was spiked with glycolic acid, G, acetic acid, A, and lactic acid, L.

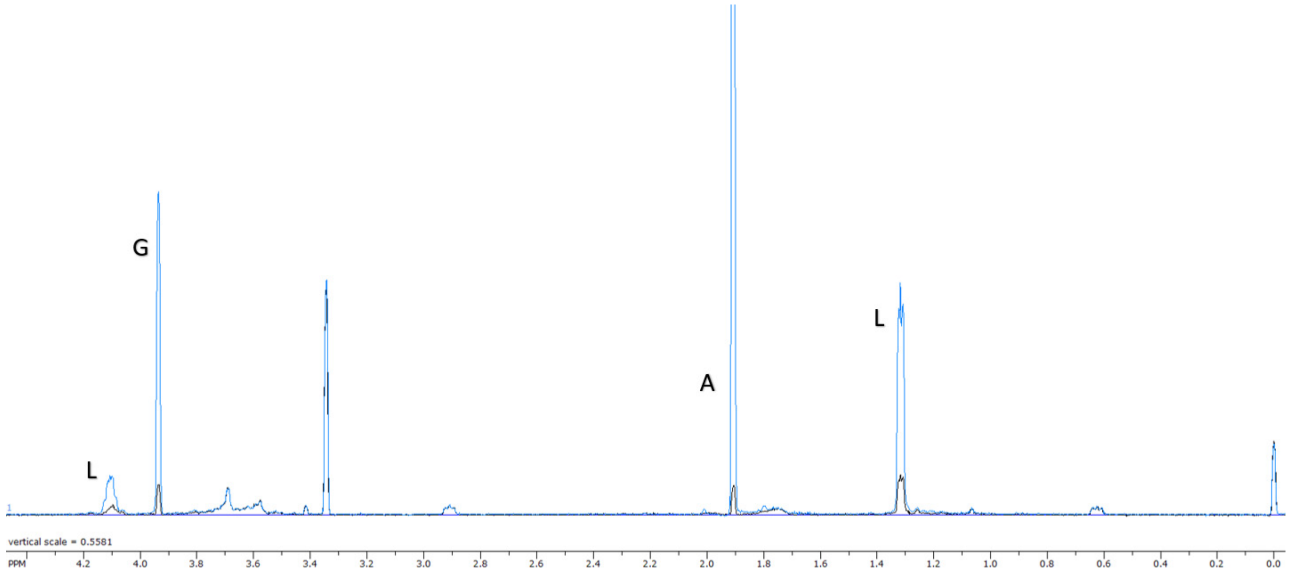

Figure S11. Spiked in-experiment. Formose solution was spiked with glycolic acid, G, acetic acid, A, and lactic acid, L (the blue spectrum). This is overlayed onto a spectrum with no spiked in products (the black baseline spectrum). Spectrum shows clear detection of glycolic acid, G, acetic acid, A, and lactic acid, L. Spectral range from 0 to 4.3 ppm.

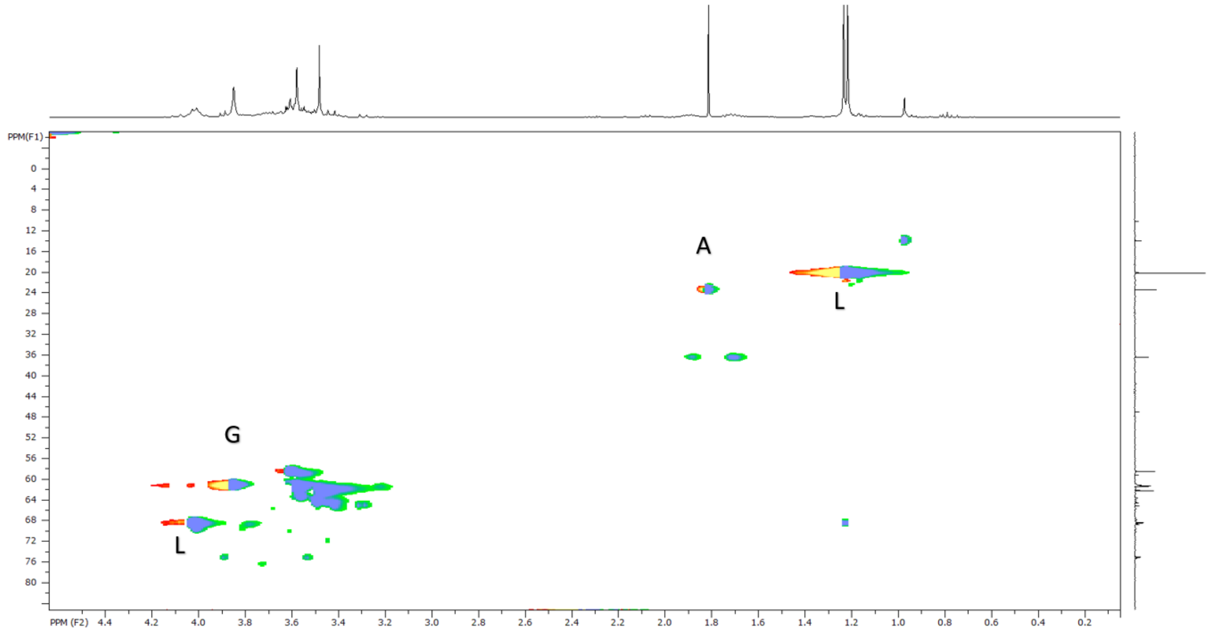

Figure S12. HSQC of C^{13} (y axis) with H^1 NMR (x axis) of a formose sample. Spectrum shows clear detection of glycolic acid, G, acetic acid, A, and lactic acid, L.

Table S1. Detected sugar values. Mass and Mol % of the glycosyl residues identified for the formose reaction dried samples.

	Samples									
	C-Sugar		Garden-Sugar		OP-Sugar		SERP-Sugar		Mag-Sugar	
Glycosyl Residues	Mass (µg)	Mol %[1]	Mass (µg)	Mol %[1]	Mass (µg)	Mol %[1]	Mass (µg)	Mol %[1]	Mass (µg)	Mol %[1]
Arabinose (Ara)	N.D.	-	2.81	15.0	N.D.	-	N.D.	-	N.D.	-
Xylose (Xyl)	Trace	27.0	9.22	49.2	Trace	37.0	Trace	22.6	Trace	53.6
Mannose (Man)	Trace	9.76	1.44	6.40	Trace	1.0	N.D.	-	N.D.	-
Glucose (Glc)	Trace	63.2	6.61	29.4	Trace	62.0	Trace	77.4	Trace	46.4
SUM[1]	Trace	100	20.1	100	Trace	100	Trace	100	Trace	100
Total Carbohydrate % by Weight	Trace	%	1.00	%	Trace	%	Trace	%	Trace	%
Mass of Sample	2 mg		2 mg		2 mg		2 mg		4 mg	

[1]Values are expressed as mole percent of total carbohydrate. The total Mol % may not add to exactly 100% due to rounding.

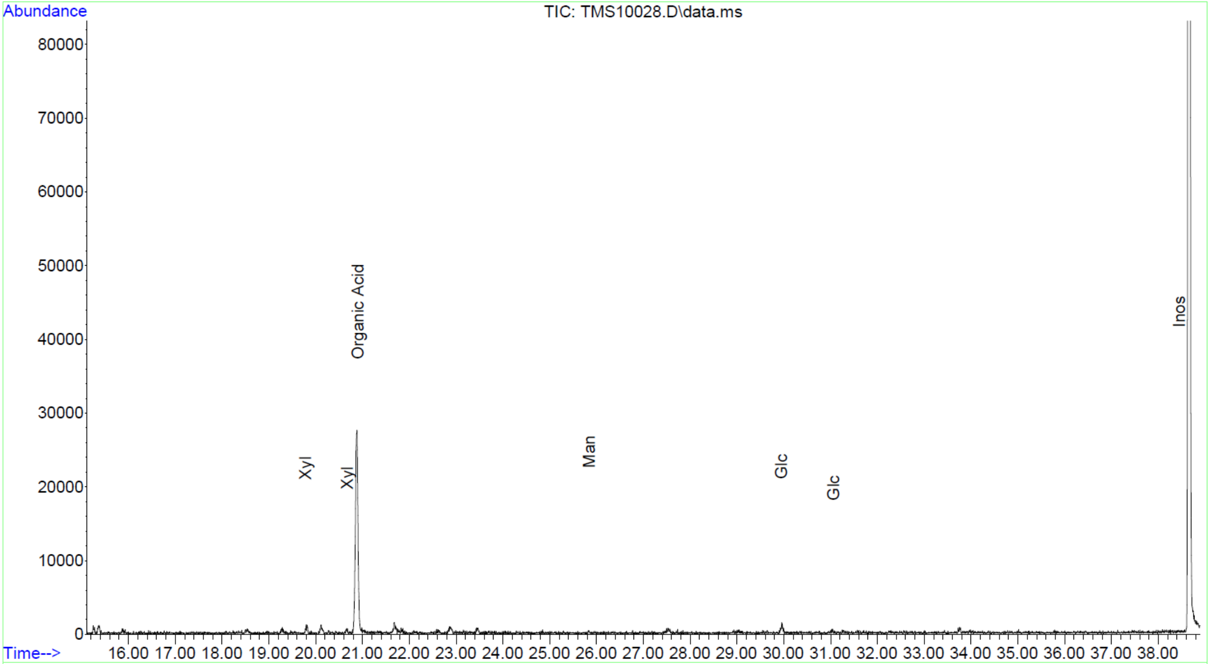

Figure S13. Gas Chromatogram for the control formose reaction which contained no minerals or chemical gardens.

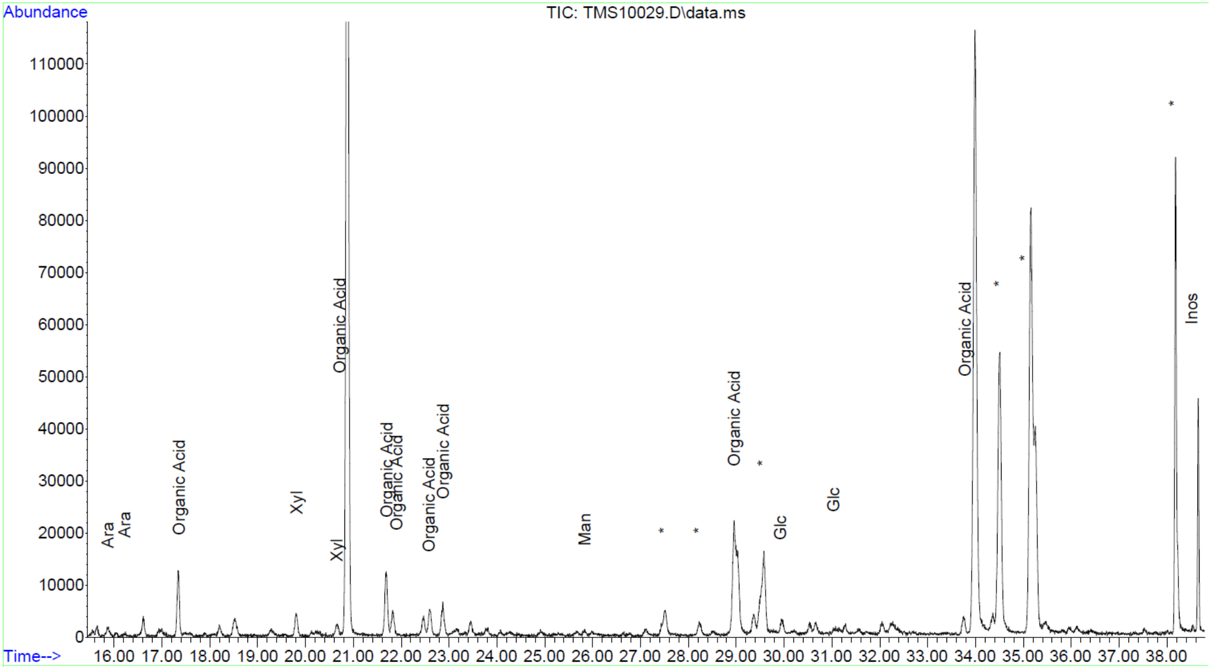

Figure S14. Gas Chromatogram for the formose reaction which contained fragments of Ca/Mg-based chemical gardens. * Represent sugar like signatures that may be branched chain sugar species.

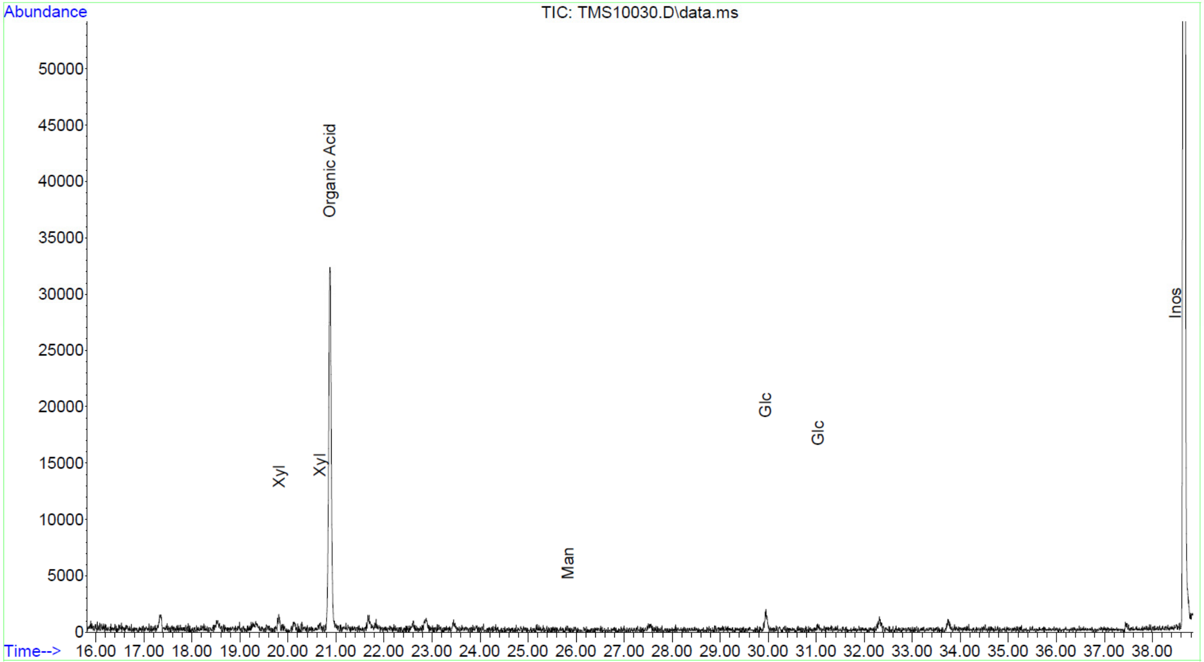

Figure S15. Gas Chromatogram for the formose reaction which contained serpentinite minerals.

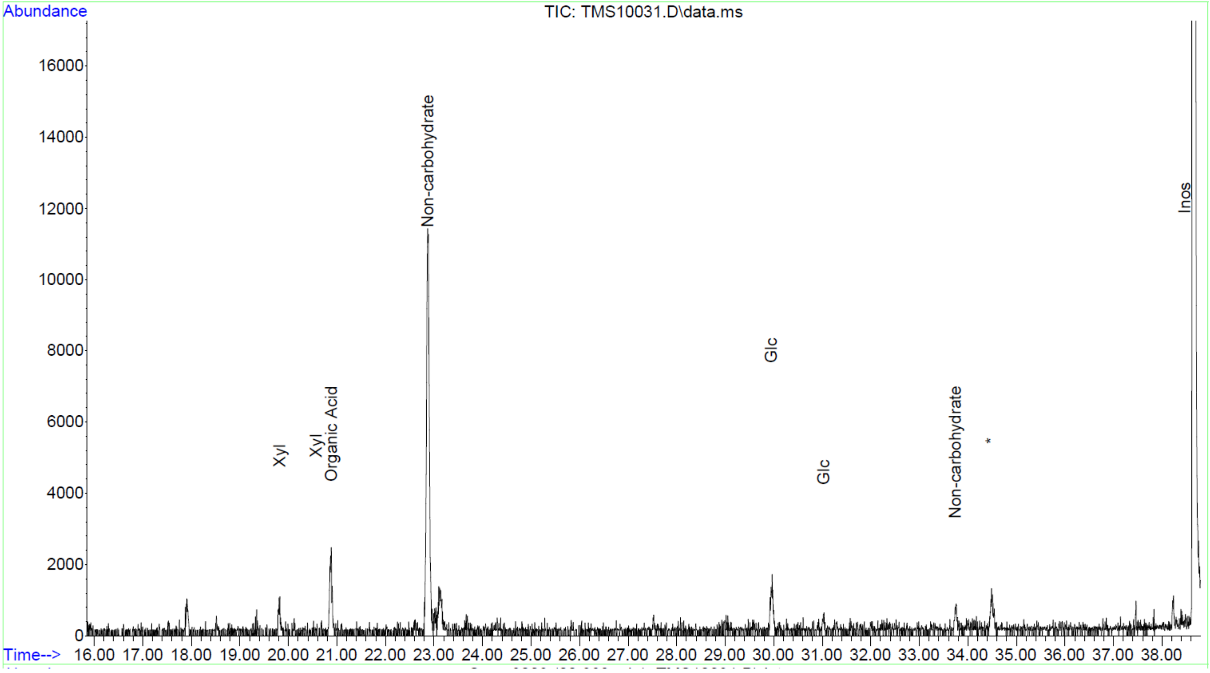

Figure S16. Gas Chromatogram for the formose reaction which contained olivine minerals.

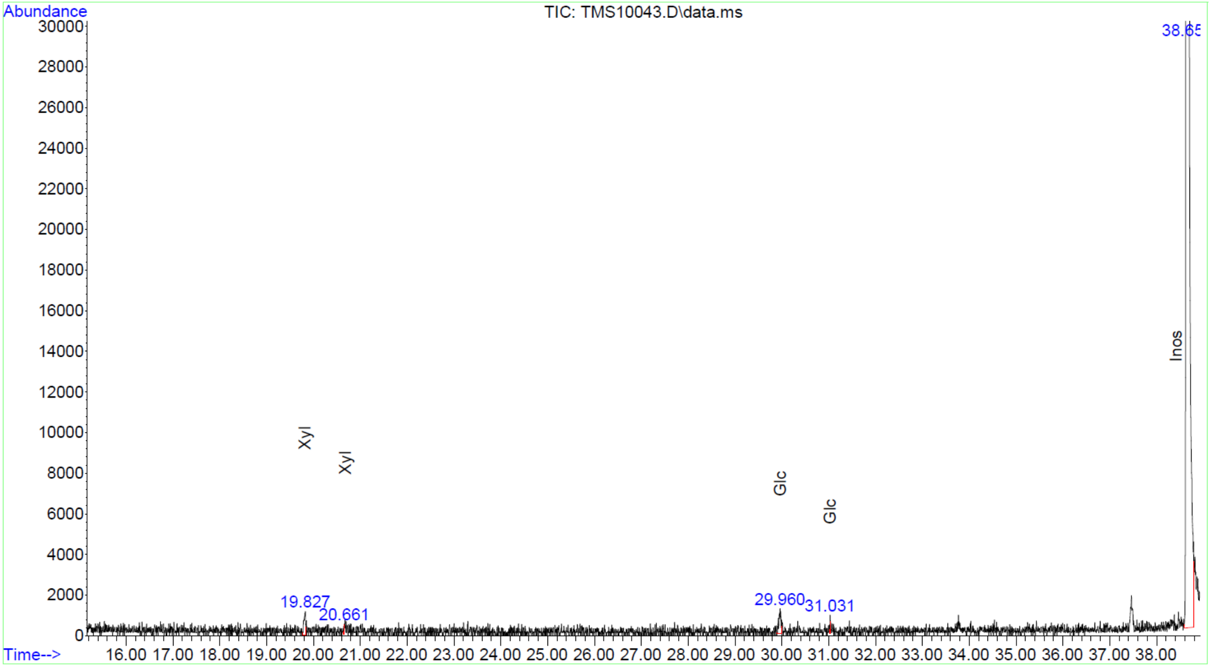

Figure S17. Gas Chromatogram for the formose reaction which contained magnetite minerals.

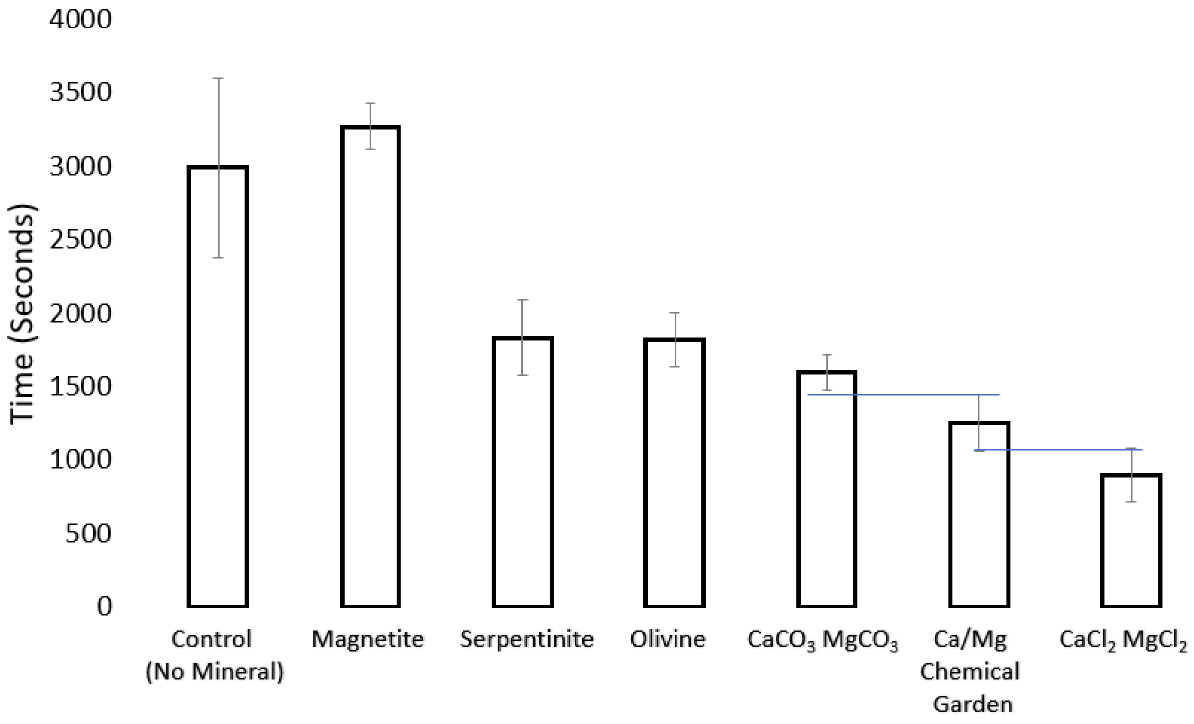

Figure S18. Observed reaction times for formose reactions in the presence of different minerals. The time it took each reaction to yellow, n=3. The chemical garden group was statistically insignificant from the soluble Ca/$MgCl_2$ group.

Table S2. Product distributions in formose reactions after 25 minutes. Data are from integral percentages.

	Formic Acid	Glycolic Acid	Lactic Acid	Acetic Acid	Methanol	Adsorbed to Mineral
Ca/Mg Chemical Garden	0.12	0.19	0.54	0.15	0.12	0.02
$CaCl_2$ $MgCl_2$	0.15	0.06	0.52	0.09	0.15	*
$CaCO_3$ $MgCO_3$	0.2	0.08	0.22	0.09	0.2	0.41
Serpentinite	0.24	0.07	0.36	0.08	0.24	0.01
Olivine	0.2	0.08	0.31	0.07	0.2	0.14
Magnetite	0.22	0	0	0	0.22	0.56
No Mineral	0.26	0	0	0	0.26	*

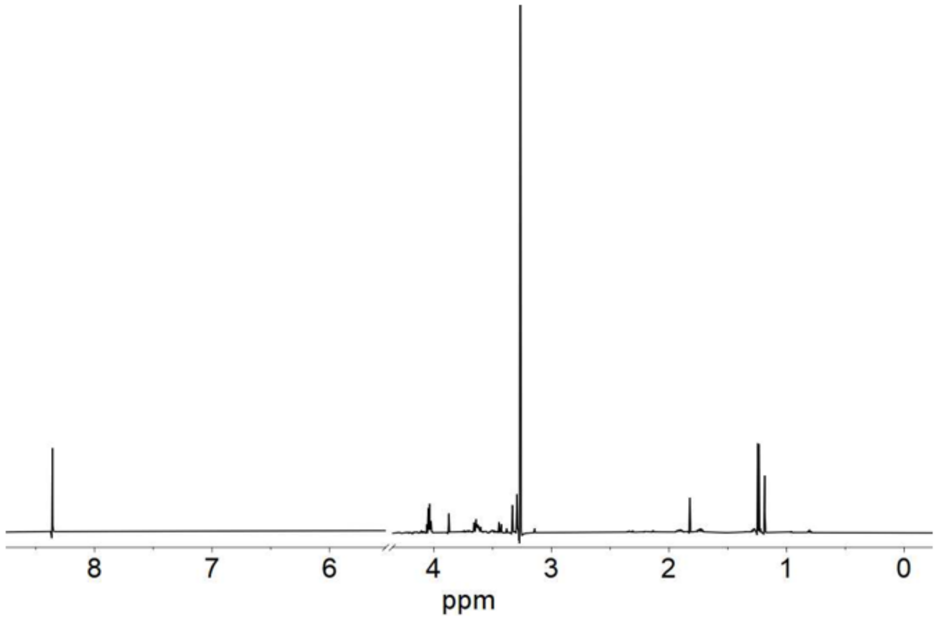

Figure S19. $CaCl_2$ $MgCl_2$ (all aqueous) formose catalyzed solution proton NMR.

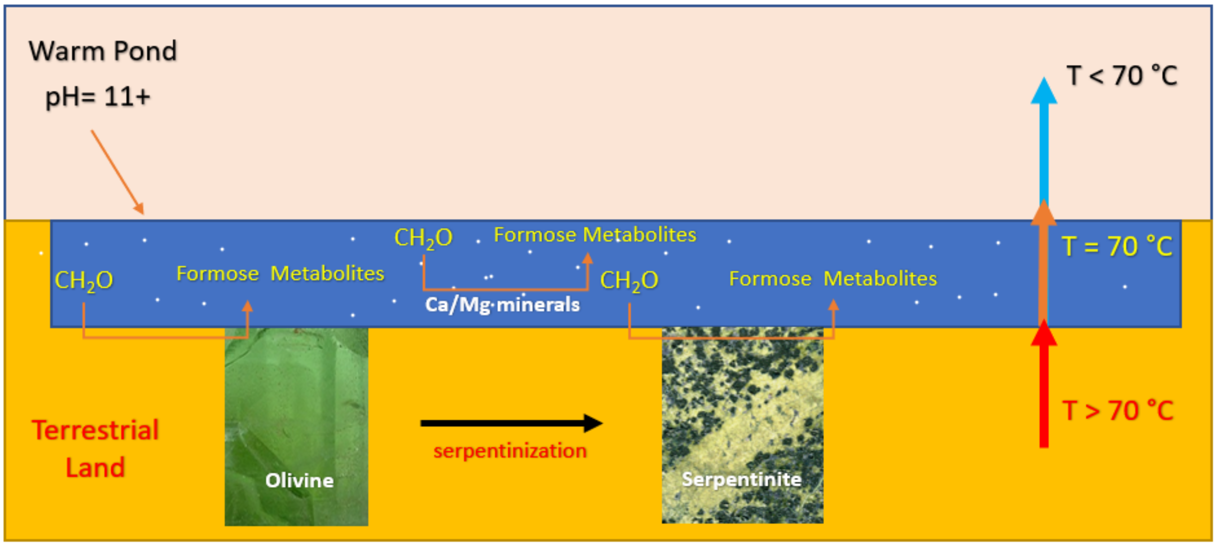

Reaction Scheme S1. A Model for a Proto Metabolic Formose System on dry land.

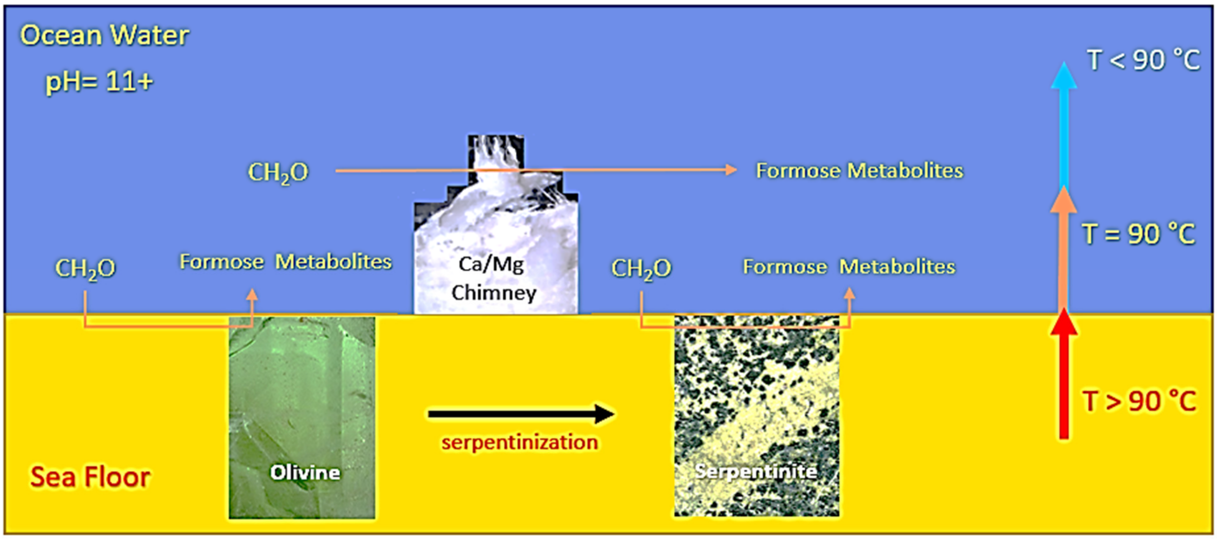

Reaction Scheme S2. A Model for a Proto Metabolic Formose System at a hydrothermal vent.

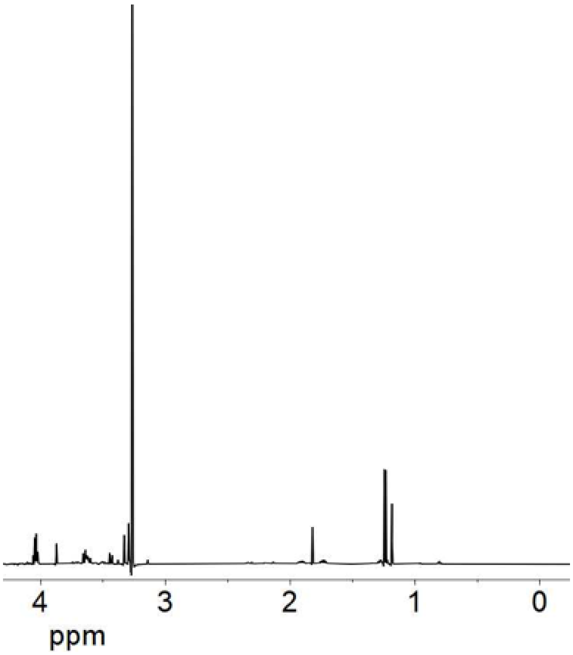

Figure S20. Sugar break down control. 0.167 Glucose solution heated at 90 C for 30 minutes at pH. 12.5 proton NMR. We see the same peaks for lactic acid as we do in our formose reactions.
